# Network Bending: Expressive Manipulation of Generative Models in Multiple Domains

**DOI:** 10.3390/e24010028

**Published:** 2021-12-24

**Authors:** Terence Broad, Frederic Fol Leymarie, Mick Grierson

**Affiliations:** 1 Department of Computing, Goldsmiths, University of London, London SE14 6NW, UK; ffl@gold.ac.uk; 2 Creative Computing Institute, University of the Arts London, London SE5 8UF, UK; m.grierson@arts.ac.uk

**Keywords:** deep generative models, expressive manipulation, active divergence

## Abstract

This paper presents the *network bending* framework, a new approach for manipulating and interacting with deep generative models. We present a comprehensive set of deterministic transformations that can be inserted as distinct layers into the computational graph of a trained generative neural network and applied during inference. In addition, we present a novel algorithm for analysing the deep generative model and clustering features based on their spatial activation maps. This allows features to be grouped together based on spatial similarity in an unsupervised fashion. This results in the meaningful manipulation of sets of features that correspond to the generation of a broad array of semantically significant features of the generated results. We outline this framework, demonstrating our results on deep generative models for both image and audio domains. We show how it allows for the direct manipulation of semantically meaningful aspects of the generative process as well as allowing for a broad range of expressive outcomes.

## 1. Introduction

**Figure 1:**
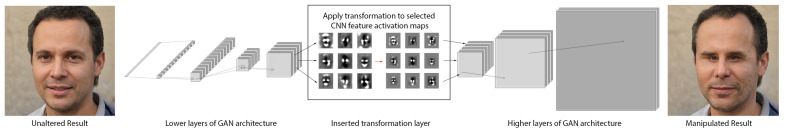
Overview of our *network bending* approach where deterministically controlled transformation layers can be inserted into a pre-trained network. As an example, a transformation layer that scales the activation maps by a factor of $k_{x}=k_{y}=0.6$ is applied (Section 5.2) to a set of features in layer 5 responsible for the generation of eyes, which has been discovered in an unsupervised fashion using our algorithm to cluster features based on the spatial similarity of their activation maps (Section 4). On the left we show the sample generated by StyleGAN2 [4] trained on the FFHQ dataset without modification, while on the right we show the same sample generated with the scaling transform applied to the selected features. NB: the GAN network architecture diagram shown in the middle of the figure is for illustrative purpose only.

The *network bending* framework [1], allows for the direct and expressive manipulation of deep generative models. First demonstrated solely for generative models in the image domain, this paper presents how *network bending* can be used in both the image and audio domains. *Network bending* allows for *active divergence* [2,3] from the original training distribution in a flexible way that provides a broad range of expressive outcomes. Our framework includes a wide array of filters that can be inserted into the network and applied to any assortment of features, in any layer, in any order. We use a plug-in architecture to dynamically insert these filters as individual layers inside the computational graph of the pre-trained generative neural network, ensuring efficiency and minimal dependencies. As this process is altering the computation graph of the model, changes get applied to the entire distribution of generated results. We also present a novel approach to grouping together features in each layer, that can be used for both image and audio domains. This clustering is based on the spatial similarity of the activation map of the features and is done to reduce the dimensionality of the parameters that need to be configured by the user, an overview of which can be seen in Figure 1. It gives insight into how *groups* of features combine to produced different aspects of the image. We show results from these processes on two kinds of generative models; using StyleGAN2, the current state-of-the-art for unconditional image generation [4], and a custom variational autoencoder (VAE) trained on spectrograms of music samples. We map out a pipeline to harness the generative capacity of deep generative models in producing novel and expressive outcomes.

## 2. Related Work

### 2.1. Deep Generative Models

A generative model consists of the application of machine learning to learn a configuration of parameters that can approximately model a given data distribution. This was historically a very difficult problem, especially for domains of high data dimensionality such as for audio and images. With the advent of deep learning and large training datasets, great advances were made in the last decade. Deep neural networks are now capable of generating realistic audio [5,6] and images [4,7,8]. In the case of images, variational autoencoders [9,10] and Generative Adversarial Networks (GANs) [11] have been major breakthroughs that provide powerful training methods. Over the past few years there has been major improvements to their fidelity and training stability, with application of convolutional architecture [12], progressively growing architecture [13], leading to the current state of the art in producing unconditional photo-realistic samples in StyleGAN [8] and then StyleGAN2 [4]. One class of conditional generative models that take inputs in the form of semantic segmentation maps can be used to perform semantic image synthesis, where an input mask is used to generate an image of photographic quality [14,15,16].

Understanding and manipulating the *latent space* of generative models has subsequently been a growing area of research. Semantic latent manipulation consists of making informed alterations to the latent code that correspond to the manipulation of different semantic properties present in the data. This can be done by operating directly on the latent codes [17,18] or by analysing the activation space of latent codes to discover interpretable directions of manipulation in latent space [19]. Evolutionary methods have been applied to search and map the latent space [20,21] and interactive evolutionary interfaces have also been built to operate on the latent codes [22] for human users to explore and generate samples from generative models.

### 2.2. Analysis of Deep Neural Networks

Developing methods for understanding the purpose of the internal features (aka hidden units) of deep neural networks has been an on-going area of research. In computer vision and image processing applications, there have been a number of approaches, such as through visualisation, either by sampling patches that maximise the activation of hidden units [23,24], or by using variations of backpropagation to generate salient image features [23,25]. A more sophisticated approach is *network dissection* [26] where hidden units responsible for the detection of semantic properties are identified by analysing their responses to semantic concepts and quantifying their alignment. Network dissection was later adapted and applied to generative models [26], by removing individual units, while using in combination a bounding box detector trained on the ADE20K Scene dataset [27]. This led to the ability to identify a number of units associated with the generating of certain aspects of the scene. This approach has since been adapted for music generation [28].

### 2.3. Manipulation of Deep Generative Models

The manipulation of deep generative models is itself a nascent area of research. An interactive interface built upon the GAN Dissection approach [26] was presented with the GANPaint framework in 2019 [29]. This allows users to ‘paint’ onto an input image in order to edit and control the spatial formation of hand-picked features generated by the GAN.

An approach that alters the computational graph of the model such that a change alters the entire distribution of results, is presented as an algorithm for “rewriting the rules of a generative model” [30]. In this approach, the weights from a single convolutional layer are used as an associative memory. Using a copy-paste interface, a user can then map a new element onto a generated output. The algorithm uses a process of constrained optimisation to edit values in the weight matrix to find the closest match to the copy-paste target. Once the rules of the weight matrix have been altered, all results from the generator have also been altered.

## 3. Base Models

To demonstrate our framework, we have used two different architectures of generative models in different data domains for analysis and manipulation. To demonstrate our method in the image domain we use StyleGAN2, analysing models trained on three separate data domains. To demonstrate our method on audio, we train a custom VAE on spectrograms trained on a dataset of varied musical genres. The two architectures are detailed in the following subsections.

### 3.1. StyleGAN2

In our experiments we used three StyleGAN2 models trained on different datasets: the Flickr Faces High Quality (FFHQ) [8], LSUN churches and LSUN cats datasets [31]. Details of the implementation of StyleGAN2 can be found in the original paper [4].

### 3.2. Spectrogram VAE

We train a variational autoencoder (VAE) [9,10] on spectrograms extracted from a custom dataset of varied musical genres, totalling 3461 audio tracks. We base our approach on previous methods for learning generative models of spectrograms [32] and melspectrograms [33] with VAEs. The tracks are randomly split up into short sequences and the Fourier transform is performed with a hop size of 256 and a window size of 1024 to produce spectrograms that have a bin size of 513. The spectrograms are then cut into shorter sequences of a window length of 128. These shortened spectrograms are then converted to decibels and then normalised for training with the VAE.

For the VAE we employ a convolutional architecture with a latent vector with dimension $\vec{v}\in R^{512}$. The encoder has 5 layers that use standard convolutions with a kernel size of 5 × 5, a stride of 2 × 2 and no padding for all of the layers. The decoder uses transposed convolutions, Table 1 lists the output resolution, kernel size, stride, and padding parameters for each of the 5 convolutional layers. A fully connected layer is used in both the encoder and decoder to interface between the convolutional layers and the latent vector. The model was trained for 50 epochs on the dataset with batch normalisation using a batch size of 64. The model was trained using the Adam optimiser [34] with a learning rate of 0.0003 and with $\beta_{1}=0$ and $\beta_{2}=0.99$.

After training it is possible to sample randomly in the latent space and then sample directly from the decoder. It is also possible to input audio sequences, both from the training set and outside of it, and produce reconstructions of the audio track mediated through the VAE model, in a method that we have previously referred to as *autoencoding* [36]. By performing this autoencoding procedure in combination with network bending, we can provide a new way of transforming and filtering audio sequences.

## 4. Clustering Features

As most of the layers in current state of the art generative models, such as StyleGAN2, have very large numbers of convolutional features, controlling each one individually would be far too complicated to build a user interface around and to control these in a meaningful way. In addition, because of the redundancy existing in these models, manipulating individual features does not normally produce any kind of meaningful outcome. Therefore, it is necessary to find some way of grouping them together into more manageable ensembles of sets of features. Ideally such sets of features would correspond to the generation of distinct, semantically meaningful aspects of the image, and manipulating each set would correspond to the manipulation of specific semantic properties in the resulting generated sample. In order to achieve this, we present a novel approach, combining metric learning and a clustering algorithm to group sets of features in each layer based on the spatial similarity of their activation maps. We train a separate convolutional neural network (CNN) for each layer of the respective generative models (the StyleGAN2 generator and the decoder of our VAE) with a bottleneck architecture (first introduced by Grézl et al. [37]) to learn a highly compressed feature representation; the later is then used in a metric learning approach in combination with the *k*-means clustering algorithm [38,39] to group sets of features in an unsupervised fashion.

### 4.1. Architecture

For each layer of both generative models, we train a separate CNN on the activation maps of all the convolutional features. As the resolution of the activation maps and number of features varies for the different layers of the model (a breakdown of which can be seen in Table 2) we employ an architecture that can dynamically be changed, by increasing the number of convolutional blocks, depending on what depth is required.

We employ the ShuffleNet architecture [35] for the convolutional blocks in the network, which is one of the state-of-the-art architectures for efficient inference in computer vision applications in terms of memory and speed. For each convolutional block we utilise a feature depth of 50 and have one residual block per layer. The motivating factor in many of the decisions made for the architecture design was not focused on achieving the best accuracy per se. Instead, we wanted a network that can learn a sufficiently good metric while also being reasonably quick to train (with 12–16 separate classifiers required to be trained per StyleGAN2 model). We also want a lightweight enough network, such that it could be used in a real-time setting where clusters can quickly be calculated for an individual latent encoding, or it could be used efficiently when processing large batches of samples.

After the convolutional blocks, we flatten the final layer and learn from it a mapping into a narrow bottleneck $\vec{v}\in R^{10}$, before re-expanding the dimensionality of the final layer to the number of convolutional features present in the layer of the respective generative model. The goal of this bottleneck is to force the network to learn a highly compressed representation of the different convolutional features in the generative model. While this invariably looses some information, most likely negatively affecting classification performance during training, this is in-fact the desired result. We wanted to force the CNN to combine features of the activation maps with similar spatial characteristics so that they can easily be grouped together by the clustering algorithm. Another motivating factor is that the clustering algorithm we have chosen (*k*-means) does not scale well for feature spaces with high dimensionality.

### 4.2. Training

We generated a training set of the activations of every feature for every layer of 1000 randomly sampled images, and a test set of 100 samples for the models trained on all of the datasets used in our experiments. We trained each CNN using the softmax feature learning approach [40], a reliable method for distance metric learning. This method employs the standard softmax training regime [41] for CNN classifiers. Each classifier has been initialised with random weights and then trained for 100 epochs using the Adam optimiser [34] with a learning rate of 0.0001 and with $\beta_{1}=0.9$ and $\beta_{2}=0.999$. All experiments were carried out on a single NVIDIA GTX 1080ti. The batch size used for training the classifiers for the various layers of StyleGAN2 can be seen in Table 2. the classifiers for the VAE were all trained with a batch size of 100.

After training, the softmax layer is discarded and the embedding of the bottleneck layer is used as the discriminative feature vector where the distances between points in feature space permit to gauge the degree of similarity of two samples. Our approach differs from standard softmax feature learning in that we use the feature vector from the bottleneck, rather than the last layer prior to softmax classification, giving a more compressed feature representation than the standard softmax feature learning approach.

### 4.3. Clustering Algorithm

Once the CNNs for every layers have been trained, they can then be used to extract feature representations of the activation maps of the different convolutional features corresponding to each individual layer of the generative model. There are two approaches to this. The first is to perform the clustering on-the-fly for a specific latent for one sample. A user would want to do this to get customised control of a specific sample, such as a latent that has been found to produce the closest possible reproduction of a specific person from the StyleGAN2 model trained on the FFHQ dataset [4,42]. The second approach is to perform clustering based on an average of features’ embedding drawn from many random samples, which can be used to find a general purpose set of clusters.

The clustering algorithm for a single example is activated by a forward pass of the generative model performed without any additional transformation layers being inserted, this to obtain the unmodified activation maps. The activation map $X_{df}$ for each layer *d* and feature *f* is fed into the CNN metric learning model for that layer $C_{d}$ to get the feature vector ${\vec{v}}_{df}$. The feature vectors for each layer are then aggregated and fed to the *k*-means clustering algorithm—using Lloyd’s method [38] with Forgy initialization [39,43]. This results in a pre-defined number of clusters for each layer. Sets of features for each layer can then be manipulated in tandem by the user.

Alternatively, to find a general purpose set of clusters, we first calculate the mean feature vector ${\vec{v}}_{df}$ that describes the spatial activation map for each convolutional feature in each layer of generative model from a set of *N* randomly generated samples—the results herein are from processing 1000 samples. Then we perform the same clustering algorithm as previously for individual samples on the mean feature vectors. The number of clusters for each layer in StyleGAN2 can be seen in Table 2. Table 1 shows the number of clusters for each layer of the decoder of the spectrogram VAE.

## 5. Transformation Layers

**Figure 2:**
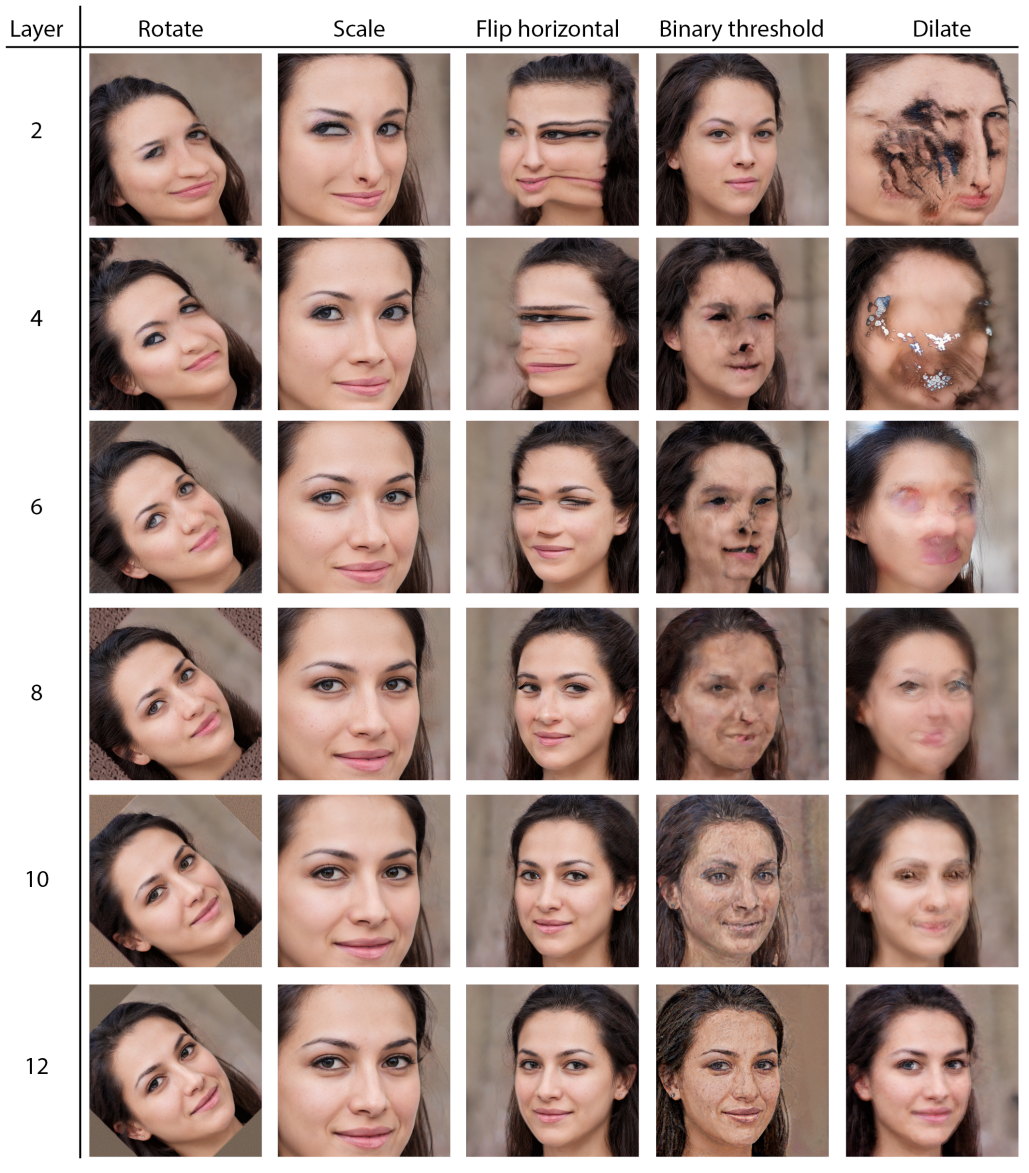
A comparison of various transformation layers inserted and applied to all of the features in different layers in the StyleGAN2 network trained on the FFHQ dataset, shows how applying the same filters in different layers can make wide-ranging changes the generated output. The rotation transformation is applied by an angle θ=45. The scale transformation is applied by a factor of $k_{x}=k_{y}=0.6$. The binary threshold transformation is applied with a threshold of t=0.5. The dilation transformation is applied with a structuring element with radius r=2 pixels.

We have implemented a broad variety of deterministically controlled transformation layers that can be dynamically inserted into the computational graph of the generative model. The transformation layers are implemented natively in PyTorch [44] for speed and efficiency. We treat the activation maps of each feature of the generative model as 1-channel images in the range −1 to 1. Each transformation is applied to the activation maps individually before they are passed to the next layer of the network. The transformation layers can be applied to all the features in a layer, or a random selection, or by using pre-defined groups automatically determined based on spatial similarity of the activation maps (Section 4). Figure 2 shows a comparison of a selection of these transformations applied to all the features layer-wide in various layers of StyleGAN2.

### 5.1. Numerical Transformations

We begin with simple numerical transformations f(x) that are applied to individual activation units *x*. We have implemented four distinct numerical transformations: the first is *ablation*, which can be interpreted as f(x)=x·0. The second is *inversion*, which is implemented as f(x)=1−x. The third is *multiplication by a scalarp* implemented as f(x)=x·p. The final transformation is *binary thresholding* (often referred to as posterisation) with threshold *t*, such that:

(1) $$f\left(x\right)=\left\{\begin{array}{cc} 1, & \mathrm{if}\;x\geq t\\ 0, & \mathrm{otherwise} \end{array}\right.$$

### 5.2. Affine Transformations

For this set of transformations we treat each activation map *X* for feature *f* as an individual matrix, that simple affine transformations can be applied too. The first two are horizonal and vertical *reflections* that are defined as:

(2) $$X\left[\begin{array}{ccc} -1 & 0 & 0\\ \;0 & 1 & 0\\ \;0 & 0 & 1 \end{array}\right]\;,\;X\left[\begin{array}{ccc} 1 & \;0 & 0\\ 0 & -1 & 0\\ 0 & \;0 & 1 \end{array}\right]$$

The second is *translations* by parameters $p_{x}$ and $p_{y}$ such that:

(3) $$X\left[\begin{array}{ccc} 1 & 0 & p_{x}\\ 0 & 1 & p_{y}\\ 0 & 0 & 1 \end{array}\right]$$

The third is *scaling* by parameters $k_{x}$ and $k_{y}$ such that:

(4) $$X\left[\begin{array}{ccc} k_{x} & 0 & 0\\ 0 & k_{y} & 0\\ 0 & 0 & 1 \end{array}\right]$$

Note that in this paper we only report on using uniform scalings, such that $k_{x}=k_{y}$. Finally, fourth is *rotation* by an angle θ such that:

(5) $$X\left[\begin{array}{ccc} cos(\theta ) & -sin(\theta ) & 0\\ sin(\theta ) & cos(\theta ) & 0\\ 0 & 0 & 1 \end{array}\right]$$

Other affine transformations can easily be implemented by designing the matrices accordingly.

### 5.3. Morphological Transformations

We have implemented two of the possible basic mathematical morphological transformation layers, performing *erosion* and *dilation* [45] when applied to the activation maps, which can be interpreted as 1-channel images. These can be configured with the parameter *r* which is the radius for a circular kernel (aka structural element) used in the morphological transformations.

## 6. Manipulation Pipeline

In our current implementation, transforms are specified in YAML configuration files [46], such that each transform is specified with five items: (i) the layer, (ii) the transform itself, (iii) the transform parameters, (iv) the layer type (i.e., how the features are selected in the layer: across all features in a layer, to pre-defined clusters, or to a random selection of features), and (v) the parameter associated with the layer type (either the cluster index, or the percentage of features the filter will randomly be applied to). There can be any number of transforms defined in such a configuration file.

After loading the configuration, we either lookup which features are in the cluster index, or randomly apply indices based on the random threshold parameter. Then the latent is loaded, which can either be randomly generated, or be predefined in latent space *z*, or be calculated using a projection in latent space *w* [4,42] (in the case of StyleGAN2). The latent code is provided to the generator network and inference is performed. As our implementation is using PyTorch [44], a dynamic neural network library, these transformation layers can therefore be inserted dynamically during inference as and when they are required, and applied only to the specified features as defined by the configuration. Once inference is unrolled, the generated output is returned. Figure 1 provides a visual overview of the pipeline, as well as a comparison between a modified and unmodified generated sample.

### Chaining Stochastic Layers

**Figure 3:**
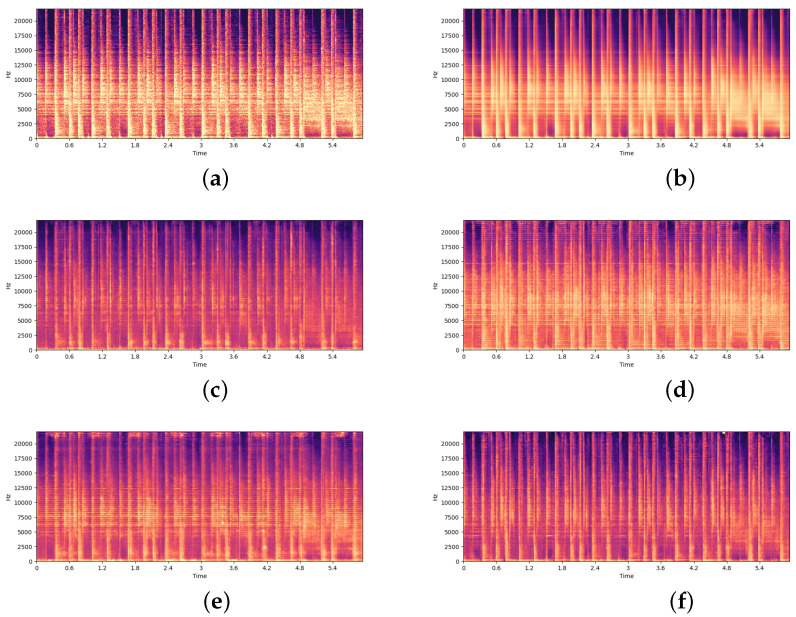
Creating stochastic variations of an audio sample by chaining stochastic transformation layers. (**a**) Spectrogram of an original source track not in the training set. (**b**) Reconstruction of source track using VAE without manipulation. (**c**–**f**) Reconstruction of the same signal using different random permutations of the same configuration, which is as follows: 25% of the features in layer 1 have been eroded with a structuring element with radius r=2 pixels, 25% of the features in layer 2 have been dilated a structuring element with radius r=2 pixels, 100% of the features in layer 3 have been filtered with the binary threshold filter with a threshold of t=0.5, 25% of the features in layer 4 have been multiplied by a factor of 1.5. Audio sample is reprinted and transformed with permission from [47]. CC0 1.0 licence.

By combining multiple stochastic layers, it is possible to create a vast number of permutations using a single configuration. Figure 3 shows that by using one configuration, many stochastic variations of an audio sample can be produced. In this example a drum break has been reconstructed using the SpectrogramVAE with a configuration applying three different stochastic transformations to 25% of the convolutional features in layers 1, 2 & 4 in combination with a layer-wide transformation being applied in layer 3. This method allows for a workflow where through experimentation a user can iteratively experiment with different configurations in an exploratory fashion until finding one that produces interesting results. Once a suitable configuration is found, a large number of stochastic variations can be produced, and then the best ones can be selected by the user. This process is one that could be particularly useful for music production, where an artist may want to create multiple variations of recordings they have created, that can later be layered into a music composition. An alternative use-case of this process used in the image domain is given in [1], where the chaining of multiple stochastic layers was used in the production of a series of five EP (extended play record) artworks that shared a common aesthetic theme.

## 7. Discussion

In this section, we discuss five perspectives: expressive manipulation, active divergence, comparisons of our results between the image and audio domains, comparisons with other methods, and finally we show some real work examples where network bending has been used in the production of artworks.

### 7.1. Expressive Manipulation

**Figure 4:**
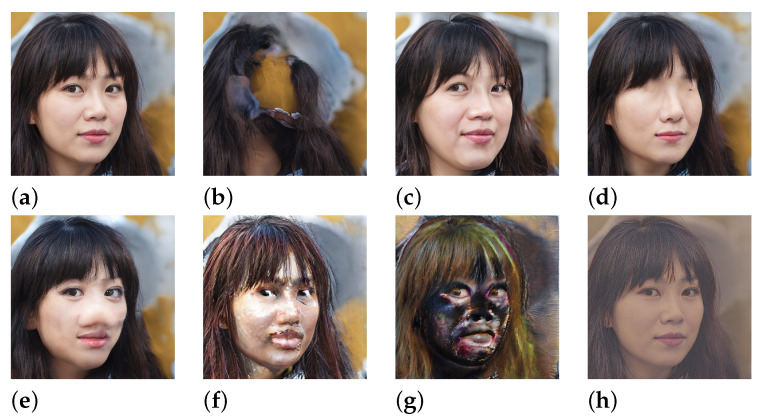
Examples from our clustering approach in the image domain. Clusters of features in different layers of the model are responsible for the formation of different image attributes. (**a**) The unmanipulated result. (**b**) A cluster in layer 1 has been multiplied by a factor of −1 to completely remove the facial features. (**c**) A cluster in layer 3 has been multiplied by a factor of 5 to deform the spatial formation of the face. (**d**) A cluster in layer 6 has been ablated to remove the eyes. (**e**) A cluster in layer 6 has been dilated with a structuring element with radius r=2 pixels to enlarge the nose. (**f**) A cluster in layer 9 has been multiplied by a factor of 5 to distort the formation of textures and edges. (**g**) A cluster of features in layer 10 have been multiplied by a factor of −1 to invert the highlights on facial regions. (**h**) A cluster of features in layer 15 has been multiplied by a factor of 0.1 to desaturate the image. All transformations have been applied to sets of features discovered using our feature clustering algorithm (Section 4) in the StyleGAN2 model trained on the FFHQ dataset.

**Figure 5:**
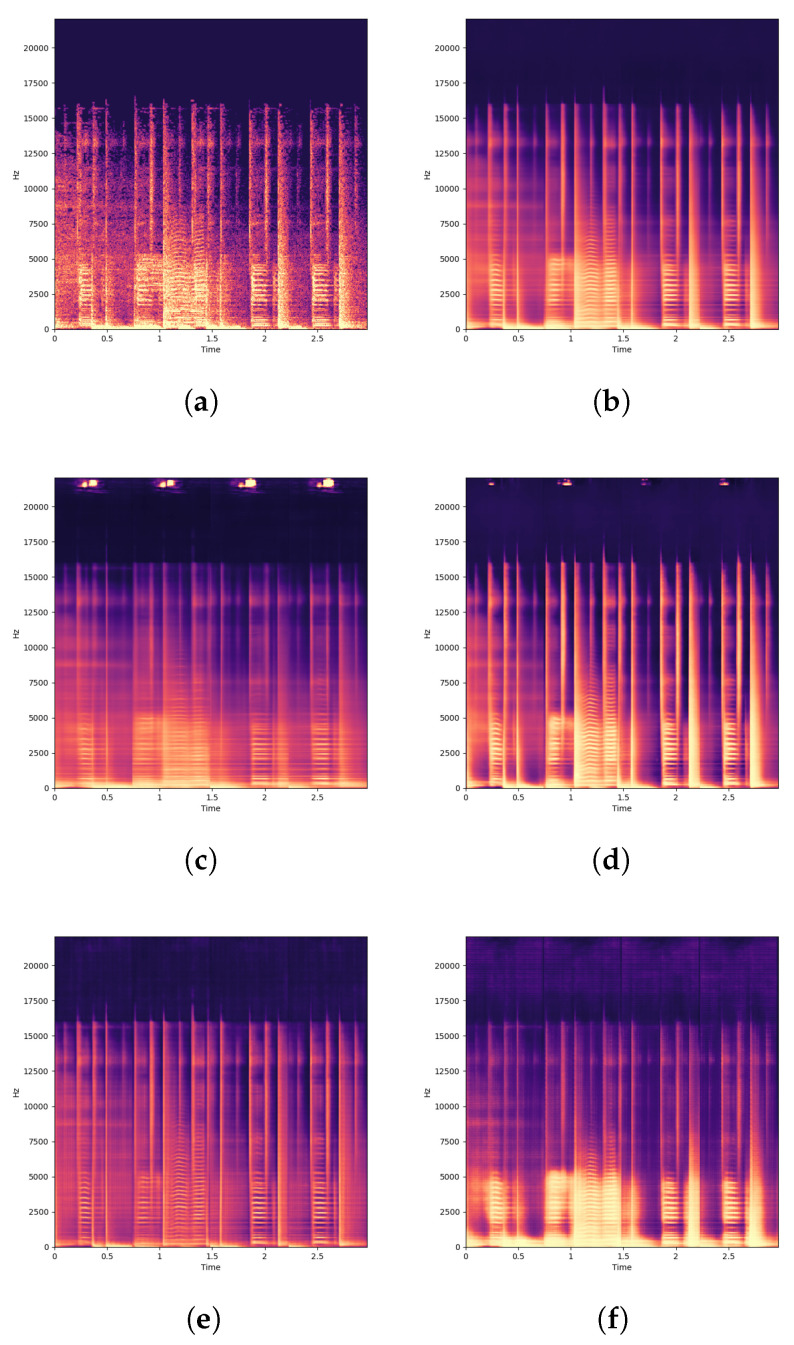
Examples from our clustering approach in the audio domain. (**a**) Spectrogram of an original source track not in the training set. (**b**) Reconstruction of source track using VAE without manipulation. (**c**) Reconstruction of the same signal where a cluster in layer 1 responsible for the generation of the transients of the signal has been ablated. (**d**) Reconstruction of the same signal where the same cluster in layer 1 responsible for the transients has been multiplied by a factor of 2, increasing the intensity of the transients in the resulting signal. (**e**) Reconstruction of the signal where a cluster in layer 3 responsible for the low and mid-range frequencies has been eroded with a structuring element with radius r=2 pixels, diminishing the intensity of these frequency components. (**f**) Reconstruction of the signal where the same cluster in layer 3 responsible for the low and mid-range frequencies has been dilated with a structuring element with radius r=2 pixels, increasing the intensity of these frequency components. Audio sample is reprinted and transformed with permission from [48]. CC BY-NC 4.0 licence.

The main motivation of the clustering algorithm presented in this paper was to simplify the parameter space in a way that allows for more meaningful and controllable manipulations whilst also enhancing the expressive possibilities afforded by interacting with the system. Our results show that the clustering algorithm is capable of discovering groups of features that correspond to the generation of different semantic aspects of the results, which can then be manipulated in tandem. These semantic properties are discovered in an unsupervised fashion, and are discovered across the entire hierarchy of features present in the generative model. For example, Figure 4 shows the manipulation of groups of features across a broad range of layers that control the generation of: the entire face, the spatial formation of facial features, the eyes, the nose, textures, facial highlights and overall image contrast. Figure 5 shows how our clustering algorithm performed in the audio domain, to demonstrate how aspects of the audio signal such as the transients and frequency components can be manipulated with various kinds of transformations.

Grouping and manipulating features in a semantically meaningful fashion is an important component for allowing expressive manipulation. However, artists are often also ready to consider surprising, unexpected results, to allow for the creation of new aesthetic styles, which can become uniquely associated to an individual or group of creators. Therefore the tool needs to allow for unpredictable as well as predictable possibilities, which can be used in an exploratory fashion and can be mastered through dedicated and prolonged use [49]. There is usually a balance between utility and expressiveness of a system [50]. While it will be required to build an interface and perform user studies to more conclusively state that our approach has struck such a balance, our current results do show that both predictable semantic manipulation and more unpredictable, expressive outcomes are possible. This is a good indication that our approach represents a good initial step, and with further refinements it can become an innovative powerful tool for producing expressive outcomes, when using deep generative models.

### 7.2. Active Divergence

One of the key motivations of our network bending approach, was to allow for the direct manipulation of generative models, in order to achieve *active divergence* from the training data [2,3]. One common criticism of using deep generative models in an artistic and creative context, is that they can only reproduce samples that *fit* the distribution of samples in the training set. However, by introducing deterministic controlled filters into the computation graph during inference, these models can be used to produce a large array of novel results. Figure 2 shows how the results vary drastically by applying the same transformation with the same parameters to different layers. Because our method alters the computational graph of the model, these changes to the results take effect across the entire distribution of possible results that can be generated. The results we have obtained markedly lie outside the distribution of training images, and allow for a very large range of possible outcomes. In addition, the combination of autoencoding [36] and network bending techniques allows for completely novel approaches to filtering and transforming pre-recorded audio, which can be seen in Figure 3.

### 7.3. Comparison between Audio and Image Domains

In this paper, we have demonstrated our network bending framework in both the image and audio domains. For the image domain we have used StyleGAN2 [4], the state of the art generative model for unconditional image generation, in the audio domain we have built our own custom generative model to demonstrate how the same principles of clustering features and applying transformations to clustered features first presented in [1] can be applied directly to another domain. The generative model for audio we have presented is building on a much smaller body of research, and has more room for improvement in terms of the fidelity of the generated outputs, however it is still adequate and demonstrates that our clustering algorithm is capable of discovering semantically meaningful components of the signal (Figure 5). Some of the transformation layers that were designed for image based models such as rotation and scaling do not transfer meaningfully into the audio domain. However, numerical and morphological transformations do work effectively in the audio domain, representing a completely new approach for manipulating audio signals.

### 7.4. Comparison with Other Methods

With respect to the semantic analysis and manipulation of a generative model, our approach of clustering features and using a broad array of transformation layers is a significant advance over previous works [26,28,29,51]. This recent thread of techniques only interrogate the function of individual features, and as such are unlikely to be capable of capturing a full account of how a deep network generates results, since such networks tend to be robust to the transformation of individual features.

**Figure 6:**
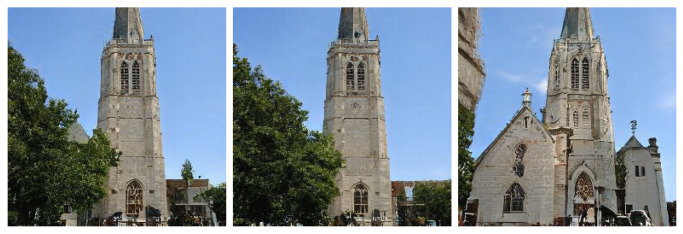
Groups of features that are not particularly sensitive to ablation may be more sensitive to other kinds of transformation. Left: original unmodified input. Middle: a cluster of features in layer 3 that has been ablated. Right: the same cluster of features that has been multiplied by a scalar of 5. As can be seen ablation had a negligible effect, only removing a small roof structure which was behind the foliage. On the other hand, multiplying by a factor of 5 removes the trees whilst altering the building structure to have gable roof sections on both the left and right sides of the church - which are now more prominent and take precedence in the generative process. Samples are taken from the StyleGAN2 model trained on the LSUN church dataset.

We also show that sets of features, which may not be particularly responsive to certain transformations, are very responsive to others. Figure 6 shows that in the model trained on the LSUN church dataset, a cluster of features, that when ablated has little noticeable effect on the result, can produce significant changes when using another transformation on the same cluster, here removing the trees and revealing the church building that was obscured by the foliage in the original result. This, we argue, shows that the functionality of features, or sets of features, cannot be understood only through ablation (which is the approach used in GAN dissection [26]), because of the high levels of redundancy present in the learned network parameters. We show that their functionality can be better understood by applying a wide range of deterministic transformations, of which different transformations are better suited to revealing the utility of different sets of features (Figure 4 and Figure 6).

Our method of analysis is completely *unsupervised*, and does not rely on auxiliary models trained on large labelled datasets (such as in [14,16,26]) or other kinds of domain specific knowledge. This approach therefore can be applied to any CNN based generative model architecture which has been trained on any dataset, as we demonstrate by using the exact same clustering method for both image and audio domains. This is of particular relevance to artist who create their own datasets and would want to apply these techniques to models they have trained on their own data. Labelled datasets are prohibitively time consuming (and expensive) to produce for all but a few individuals or organisations. Having a method of analysis that is completely unsupervised and can be applied to unconditional generative models is important in opening up the possibility that such techniques become adopted more broadly.

The framework we have presented is the first approach to manipulating generative models that focuses on allowing for a large array of novel expressive outcomes. In contrast to other methods that manipulate deep generative models [29,30], our approach allows the manipulation of any feature or set of features in any layer, with a much broader array of potential transformations. By allowing for the combination of many different transformations, it is evident that the outcomes can diverge significantly from the original training data, allowing for a much broader range of expressive outcomes and new aesthetic styles than would be possible with methods derived from semantic image synthesis [14,15,16] or semantic latent manipulation [17,18,19].

### 7.5. Network Bending in Practice

Since we introduced it, network bending has been used in the production of a number of artworks. The artist Derrick Schulz utilises network bending frequently in their practice of chaining models, where multiple generative models and deep learning based manipulation techniques are used in sequence to produce desired results [3]. For instance, to make the work *You Are Here* [52], Schultz chains multiple techniques including: a custom unconditional GAN, network bending, custom image translation models, and super-resolution.

**Figure 7:**
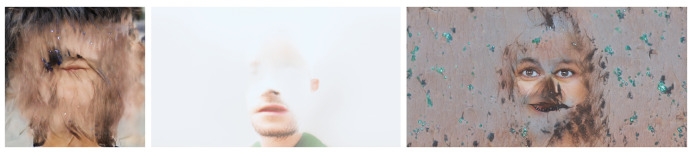
Three illustrative samples from using network bending in the production of artworks. Left: an image from the series of artworks *Teratome* [53]. Middle: a still from the video piece *Fragments of Self* [55]. Right: a still from the video piece *Disembodied gaze* [54]. Images reproduced with permission from the copyright holder.

Figure 7 shows three examples of artworks made using network bending techniques applied to the official StyleGAN2 FFHQ model. The series of artworks *Teratome* [53] is obtained by using stochastic network bending transforms to disrupt the image formation process at its very earliest incarnation in the highest layers of StyleGAN2, to produce highly detailed imagery from the corrupted formations. This results in images that have the photo-realistic qualities of portraits, but with impossible distortions and formations. The video piece *Fragments of Self* presents a self portrait (achieved by projecting a photograph into the StyleGAN2 FFHQ latent space [4,42]) that violently oscillates in and out of recognition, leaving only traces of likeness. This is achieved by ablating the convolutional features of the second layer of the model using a predetermined sequence calculated using the Perlin noise algorithm that is used to determine which of the 512 features in the convolutional layer are ablated at any given frame in the video sequence. *Disembodied gaze* [54] is a video piece that demonstrates what can be achieved by utilising the clustering method presented in this paper. The cluster of features in layer 5 that represent eyes, when ablated lead to the eyes not being generated and the model contextually fills in the blank area with skin (as can be seen in Figure 4d). To make *Disembodied gaze*, all of the clusters in layer 5 other than the cluster that generates eyes have been ablated, leaving the eyes perfectly generated but the surrounding areas are textural field of features that have the appearance of hair and skin. The video piece is composed by performing a latent space interpolation between the various identities that are generated by the FFHQ model.

## 8. Conclusions and Future Work

In this paper, we have introduced a novel approach for the interaction with and manipulation of deep generative models that we call *network bending*, which we have demonstrated on generative models in the image and audio domains. By inserting deterministic filters inside pre-trained networks, we present a framework for performing manipulation inside the networks’ black-box and utilise it to generate samples that have no resemblance to the training data, or anything that could be easily created using conventional media editing software. We also present a novel clustering algorithm that is able to group sets of features, in an unsupervised fashion, based on spatial similarity of their activation maps. We demonstrated that this method is capable of finding sets of features that correspond to the generation of a broad array of semantically significant aspects of the generated results in both image and audio domains. This provides a more manageable number of sets of features that a user could interact with.

We have demonstrated that network bending is a framework that is sufficiently expressive and flexible that it has been used in different ways in the production of a number of artworks. We have shown how this framework can be utilised for creative expression in various workflows: either by controlled direct manipulation over specific semantic properties, or in an exploratory fashion by chaining multiple stochastic transformation layers. These different approaches can be used in both the audio and image domains.

The inserting of deterministic filters into pre-trained models, has been adopted and utilised in the development and evaluation of the next generation of generative models, namely StyleGAN3 [56], which has been designed such that their internal representations are fully equivariant to either translation or rotation. This has been done in order to design models that are better suited for post-training manipulation that can be used for producing video and animations, adding weight to our claim that network bending is an important new approach to media creation with generative deep learning.

In future work we look to further advance our network bending framework in the audio domain (alongside existing parallel efforts [57,58]). We intend to do this by extending this framework to non-CNN based generative model architectures, such as sequential, autoregressive, and transformer based architectures. We also plan to extend our work into further domains such as those that produce text, video or 3D images and meshes. Finally we look to build an interface around our network bending framework and aim to better understand how artists would want to use it in their practice.

## Figures and Tables

**Table 1:** Table shows resolution, number of features of each layer, convolutional kernel size, strides, padding parameters for the decoder network in the spectrogram VAE. The last two columns on the right show the number of ShuffleNet [35] convolutional blocks for each CNN model used for metric learning, and the number of clusters calculated for each layer using *k*-means.

Layer	Resolution	#Features	Kernel Size	Stride	Padding	CNN Depth	#Clusters
1	8 × 33	512	5 × 5	1 × 2	0 × 2	1	5
2	17 × 65	256	3 × 5	2 × 2	2 × 2	2	5
3	32 × 129	128	4 × 5	2 × 2	2 × 2	3	4
4	64 × 257	64	4 × 5	2 × 2	2 × 2	4	4
5	128 × 513	1	4 × 5	2 × 2	2 × 2	-	-

**Table 2:** Table shows resolution, number of features of each layer, the number of ShuffleNet [35] convolutional blocks for each CNN model used for metric learning, the number of clusters calculated for each layer using *k*-means and the batch size used for training the CNN classifiers for the StyleGAN2 models. Note: LSUN church and cat models have only 12 layers.

Layer	Resolution	#Features	CNN Depth	#Clusters	Batch Size
1	8 × 8	512	1	5	500
2	8 × 8	512	1	5	500
3	16 × 16	512	2	5	500
4	16 × 16	512	2	5	500
5	32 × 32	512	3	5	500
6	32 × 32	512	3	5	500
7	64 × 64	512	4	5	200
8	64 × 64	512	4	5	200
9	128 × 128	256	5	4	80
10	128 × 128	256	5	4	80
11	256 × 256	128	6	4	50
12	256 × 256	128	6	4	50
13	512 × 512	64	7	3	20
14	512 × 512	64	7	3	20
15	1024 × 1024	32	8	3	10
16	1024 × 1024	32	8	3	10

## Data Availability

The generated datasets of activation maps have been made publicly available and can be found at: https://github.com/terrybroad/network-bending, accessed on 16 December 2021.
